# Changes in the Framing of Antimicrobial Resistance in Print Media in Australia and the United Kingdom (2011–2020): A Comparative Qualitative Content and Trends Analysis

**DOI:** 10.3390/antibiotics10121432

**Published:** 2021-11-23

**Authors:** Chris Degeling, Victoria Brookes, Tarant Hill, Julie Hall, Anastacia Rowles, Cassandra Tull, Judy Mullan, Mitchell Byrne, Nina Reynolds, Olivia Hawkins

**Affiliations:** 1 Australian Centre for Health Engagement, Evidence and Values, The Faculty of Arts, Social Sciences and Humanities, University of Wollongong, Wollongong, NSW 2522, Australia; juliehal@uow.edu.au (J.H.); ohawkins@uow.edu.au (O.H.); 2 Sydney School of Veterinary Science Faculty of Science, The University of Sydney, Sydney, NSW 2006, Australia; victoria.brookes@sydney.edu.au; 3 Illawarra-Shoalhaven Local Health District, NSW Health, Warrawong, NSW 2502, Australia; tarant.hill@health.nsw.gov.au; 4 School of Health & Society, The Faculty of Arts, Social Sciences and Humanities, University of Wollongong, Wollongong, NSW 2522, Australia; ajr450@uowmail.edu.au (A.R.); cassandra.tull@hotmail.com (C.T.); 5 Centre for Health Research Illawarra Shoalhaven Population, Faculty of Science, Medicine and Health, University of Wollongong, Wollongong, NSW 2522, Australia; jmullan@uow.edu.au; 6 College of Human and Health Sciences, Charles Darwin University, Darwin, NT 0909, Australia; mitchell.byrne@cdu.edu.au; 7 School of Business, Faculty of Business and Law, University of Wollongong, Wollongong, NSW 2522, Australia; ninar@uow.edu.au

**Keywords:** antibiotic resistance, newspapers, content analysis, public policy, public awareness

## Abstract

Educating the public about antimicrobial resistance (AMR) is considered a key part of an optimal public health response. In both media depictions and policy discourses around health risks, how a problem is framed underpins public awareness and understanding, while also guiding opinions on what actions can and should be taken. Using a mixed methods approach we analyse newspaper content in Australia and the United Kingdom (UK) from 2011 to 2020 to track how causes, consequences and solutions to AMR are represented in countries with different policy approaches. Analyses demonstrate greater variability in the frames used in UK newspapers reflecting large hospital and community outbreaks and a sustained period of policy reform mid-decade. Newspapers in Australia focus more on AMR causes and consequences, highlighting the importance of scientific discovery, whereas UK coverage has greater discussion of the social and economic drivers of AMR and their associated solutions. Variations in the trends of different frames around AMR in UK newspapers indicate greater levels of public deliberation and debate around immediate and actionable solutions; whereas AMR has not had the same health and political impacts in Australia resulting in a media framing that potentially encourages greater public complacency about the issue.

## 1. Introduction

Antimicrobial resistance (AMR) is an existential threat to modern medicine and broader society [1,2,3]. The key concern is that infections with resistant microbes are more difficult to treat, magnify morbidity and have much higher death rates. AMR also increases the economic cost of effective healthcare because of the need for more expensive drugs and longer hospital stays [4]. Responding to this critical and emerging public health problem became of great importance to governments, transnational organisations, and policy-makers at the turn of the century [5]. Political interest increased markedly in 2012 and 2013 following key reports from the World Economic Forum, the US Centres for Disease Control and the Chief Medical Officer for England [6,7,8]. Human healthcare and agriculture have been the focus of AMR-related policymaking [6,9,10]. As a global health concern, the key strategies for managing AMR are to reduce antimicrobial use, stop the transmission of resistant microbes within and between human and animal populations, and improve the drug development pipeline [3,7,11]. National differences in antibiotic consumption rates do not clearly correspond to the prevalence of bacterial infections [12,13]. They are related to national and local policies, accepted modes of treatment, the types of health-care and agricultural systems, and a number of cultural factors such as public trust in governments and institutions and risk aversion [14,15,16,17]. The complexity of AMR and the extent to which antibiotics and other antimicrobials have become embedded into the systems and structures that support our societies over the last 80 years mean that AMR is a problem for which there is no simple or cost-free solution [18,19,20].

Policy approaches to the problem of AMR in the United Kingdom (UK) and Australia have overlapping histories and trajectories [6]—with the former leading in policy action and practice reform (Supplementary Table S1). Since 2010, government planning to address AMR in both countries has been articulated around One Health calls for cross-sectoral collaboration, and the instantiation of new forms of surveillance, measures to reduce antimicrobial use, and incentives to increase the supply of new antimicrobial agents [21,22]. The UK Five Year Antimicrobial Resistance Strategy 2013–2018 [23] placed the national government and implicated organisations at the centre of the response to AMR, with subsequent reforms driving the abandonment of longstanding policy processes driven by expert and stakeholder committees with prioritisation of an evidence-based approach to addressing the problem [6]. In 2014, the UK government mandated AMR outcome measures and targets for implicated institutions such as healthcare and agriculture, supported by research funding for biomedical and behavioural solutions to educate and engage both professionals and publics in changing the culture around antibiotic use [24,25]. Australia adopted similar policy priorities, surveillance structures and organisational systems in healthcare with the release of Australia’s First National Antimicrobial Resistance Strategy 2015–2019 [22,26]. Both jurisdictions have since updated accreditation standards, educational materials, and curricula for healthcare providers. The UK has also taken a much more directive approach than Australia to changing antibiotic use in primary care with GPs being provided with detailed localised data on prescribing and antibiotic resistance, coupled to financial incentives to drive improvements in prescribing practices [27]. However in Australia, rather than incentivise or force change on key stakeholders, such as GPs and agricultural industries, the driver of efforts for further reform have remained tied to expert and stakeholder consensus around voluntary changes in antibiotic use [6]. Whereas public awareness of AMR in the UK is monitored, Australian governments did not provide significant resources for engagement with and monitoring of public awareness of AMR [28]. The UK [29] and Australian [30] governments have recently released updated National Action Plans with the former placing an explicit focus on actions to further reduce overprescribing in primary care, whereas the Australian plan continues to be oriented towards building on existing structures to minimise the impacts of AMR.

In concrete terms, the need to address the drivers of AMR is frequently operationalised as requiring behaviour change from clinicians, patients, and, in the case of agricultural systems, livestock producers and consumers [31,32]. Therefore, enhancing the understanding of prescribers, farmers and members of the broader public of AMR is considered to be an important component of an effective and optimal public health response [33,34]. For members of the public, news media reporting on emerging risks are important sources of scientific and health information [35]. Fragmentation in audiences means that an increasing number of people access news content through social media; but over 75% of stories they are exposed to come from news websites [36]. Therefore, news media representations of AMR remain important because they guide the way that many members of the public define and understand the problem, foreground particular causes, solutions, or both, and assign blame [37,38]. It is increasingly recognised that successfully addressing AMR requires a whole of society solution, and both national and intergovernmental health agencies continue to make statements as to the immediacy of the risks and the need for action [7,10]. In 2016, the O’Neill report (commissioned by the UK government and the Wellcome Trust in 2014) included an international call for all governments to promote greater public awareness through greater engagement with news and social media activity [39].

Previous studies of how news media represent AMR in Australia and the UK indicate that most articles are factually accurate. However, for editorial reasons, or to draw their audience’s attention to a particular feature of a news item, information relevant to AMR in these reports is presented inconsistently and with omissions [40,41]. One of the impacts of media reporting of AMR is to contribute to the fragmentation of public discourses such that they slide between apportioning blame to antibiotic prescribers and antibiotic users (loosely construed as the broader community) [33,42]. Analyses of UK media coverage of AMR between 2000 and 2015 highlight how the issue became highly politicised in this jurisdiction with scant representation of the role of agricultural industries in driving resistance or the lack of a response by pharmaceutical companies to this emerging threat [42,43]. Concomitantly, simplistic and polarised understandings of AMR as either a looming catastrophe, or, more narrowly, a problem of “dirty hospitals” have dominated political and public discussions in the UK, such that scientists and experts struggled to communicate more nuanced understandings and solutions [44,45]. In the Australian context, an analysis of the metaphors employed in major newspapers to explain AMR over the last two decades, found that reporting tends to give “superbug’ resistant microbes malign agency, around which military metaphors and doomsday scenarios are frequently employed to explain the significance of AMR to the general public [41]. A more recent analysis of major Australian print and online news media and free to air news and current affairs broadcasts in the year 2017, found the locus for action in coverage of AMR is generally placed on the need for scientific discovery and the development of further technical fixes to an emerging problem [40]. The more recent turn to science and its discoveries may indicate that the newsworthiness of the AMR crisis has passed and that stories now exercise reassurance. As a consequence the media discourse around AMR tends to portray the general public a passive bystanders, rather than as actors who need to engage in developing a collective response and take responsibility for helping to reduce antibiotic misuse [40,41].

To our knowledge, the influence of government policies on news-media coverage of AMR has not been analysed. The UK has positioned itself as a global leader in taking action against AMR, whereas the approach in Australia has been more consensus driven and cautious [6,21]. In this article, using the UK and Australia as case examples, our aim was to track the public circulation and prominence of key ideas about AMR in these countries in the last decade. We sought to map how media representations of AMR in these settings responded to changes in national AMR polices, governing structures and local contexts and events. Media discourses are also forums for political contests as experts, scientists, political leaders, advocates, policy makers, and other stakeholders can challenge each other’s views on the key causes of AMR risks and how best to manage them [46]. Understanding and directly comparing the public positioning of implicated institutions, how they have varied over time, and reflect the goals of key global and local national AMR stewardship initiatives provides context to the growing volume of critiques of AMR media and public engagement strategies [34,39,47,48]. On a broader scale, as greater attention is being paid to efforts at AMR governance and the critical importance of social and economic reforms is increasingly recognised [49,50], better knowledge of how public discourses about AMR have evolved over the last decade in countries with different policy approaches and settings can contribute to efforts to improve public engagement with and participation in future stewardship efforts [7,33].

## 2. Materials and Methods

Drawing on the methodologies used in recent analysis of representations of AMR in North American newspapers [46], we used a two-step mixed methods approach comprised of a qualitative phase in which codes are inductively developed (where the codes are derived from the data) and assigned to articles reflecting their content, followed by a quantitative analysis of frequencies of these codes, and the relationships between them [51,52]. Informed by the categories developed and deployed in previous examinations of AMR in news media [40,41,44,46,53], our inductive coding and subsequent analysis was guided by the following research questions:

1. Which types of interactions between microbes, antibiotics and people are highlighted in print media coverage?
2. Which species of antibiotic resistant microbes get media attention?
3. What reasons are given for members of the general public to care about AMR?
4. Who or what are portrayed as being at risk of AMR?
5. Who or what is portrayed as being responsible for causing AMR?
6. Who or what is portrayed as being responsible for addressing AMR?
7. What solutions to the problem of AMR are described?
8. Which stakeholders and expert groups get to speak and share their perspective on AMR in print media coverage?

To identify Australian and UK coverage in newspaper print media of issues surrounding AMR the databases *Factiva* and *Proquest Central* were searched independently for each setting using the following terms: (antimicrobial resistan*) or (super bug*) or (superbug*) or (Flesheating bacteria) or (flesh eating bacteria) or (flesh-eating bacteria) or (golden staph*) or (MRSA) for the period 1 January 2010 through 31 December 2020. After limiting content to English language and the regions of Australia or the UK, once the *Factiva* and *Proquest* samples for each setting were combined, a total of 2041 items were identified in Australian newspapers and 3698 items in the UK (See Supplementary Figure S1: PRISMA Diagram). In the first screening exercise 972 and 873 articles were excluded from the Australian and UK newspaper samples, respectively, because they were: duplicates; incomplete articles; or not published in an Australian or UK newspaper. Full-text reports of the remaining 1069 (Australia) and 2825 (UK) articles were then downloaded into an Endnote database. For the Australian sample a further 475 articles were excluded after Level 2 title review and Level 3 full text screening because the content was a duplicate or syndicated republication (128), or not relevant to AMR-related issues (347). For the UK sample, 1805 articles were excluded at level 2 and level 3 review because the content was a duplicate (1008) or not relevant to AMR-related issues (618). At the end of this process 594 unique articles in the Australian media sample and 1020 articles from the UK remained to be analysed.

The two media samples were then read, catalogued manually, and cross-compared by TH, CT, OH and C.D. to inductively identify the key categories and codes and track prominent concepts, guided by the research questions. Next, these authors manually cross-coded a pilot sample of both the UK and Australian media samples (*n* = 50 in each) to test and confirm the inter-observer reliability of the coding framework and to extend the preliminary thematic analysis. Once the face validity of the coding frame was established the remaining articles in each sample were coded as independent sets by TH, C.D., JH, AP and CT (the final codes and their definitions can be found in Supplementary Table S2). Regular discussions among the authors served to generate additional questions and hypotheses, and to validate insights as they emerged. The results from coding were then tabulated in matrix form and displayed visually as descriptive statistics in charts to aid interpretation. Charts were combined using smoothed averages of the relative proportions of each coded category reported annually, to produce comparative time-series representing the relative strength of themes throughout the study period.

## 3. Results

**Figure 1:**
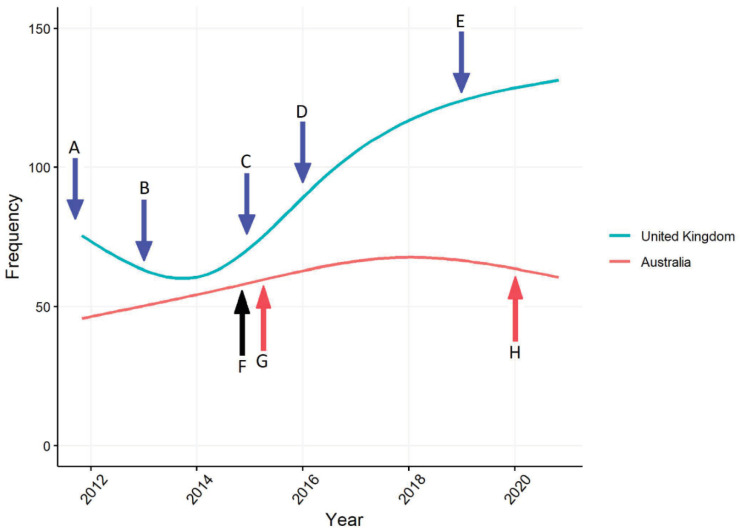
Frequency of articles on AMR in Australia and UK between 2011 and 2020. Figure key: (A) Annual Report of the Chief Medical Officer, Vol 2, 2011 [8]; (B) UK Five Year AMR Strategy 2013–18 [23]; (C) UK AMR Funder’s Forum established; (D) The O’Neil Review on Antimicrobial Resistance 2014 [11]; (E)—The UK’s Five-year National Action Plan. 2019 [29]; (F) The WHO Global Action Plan [56]; (G) Australia’s First National Antimicrobial Resistance Strategy 2015–2019 [26]; (H) Australia’s National Antimicrobial Resistance Strategy—2020 and beyond [30].

The 594 articles in the Australian sample were from 24 different mastheads—three with national circulation, seven major provincial newspapers (four tabloids and three broadsheets) and 14 regional papers from all eight States and Territories. Figure 1 shows that articles about AMR per year in Australian Newspapers peaked in 2017 but are now declining as has been found in previous studies [41]. The 1732 articles in the UK sample were from 25 different mastheads—nine broadsheets and 16 tabloids (including four freesheets), 18 with national circulation (predominantly published in London), but also papers based in Glasgow, Dundee and Blackburn. The number of articles about AMR per year in UK newspapers increased rapidly in the middle of the decade, around the same time as the UK government established the UK Medical Research Council-led Antimicrobial Resistance Funders’ Forum and ‘declared war’ on AMR in 2014 [54]. Differences in the number of articles about AMR in each setting in comparable numbers of media outlets can be explained by the strong local focus of the 14 regional newspapers in the Australian sample, consumers of which typically turn to national news-sources such as the Australian Newspaper or Australian Broadcasting Corporation (ABC) for national and international news [55].

In what follows we describe and compare the key trends in how newspapers in Australia the UK have framed the problem of AMR between 2011 and 2020. The results are presented across seven dimensions or framing categories that capture the range of ways in which media reporting can convey information to members of the public about the significance, causes, consequences and potential solutions to AMR (Supplementary Table S3). These key thematic categories and the explanations which they embody can be abstracted from news texts and shown to impact upon audience understanding [57]. For the framing categories where a significant number of options were developed during the coding process, we limit our analyses to clear trends, patterns and differences rather than minor variations. In each of the dimensions/categories reported below, where possible, we also draw on the data to contextualise the use of a specific frame with reference to events and changes in AMR policies and related practices in the 2 study settings.

### 3.1. Different Framings of the Role and Relative Importance of Microbes and Antibiotics in AMR

**Figure 2:**
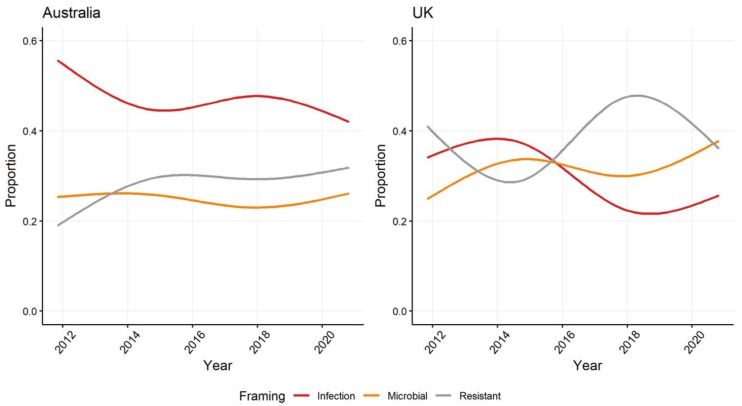
Proportion of different framings in Australian and UK newspapers of the role and importance of microbes in AMR.

Of the 594 articles in the Australian sample 571 (96%) included a frame that highlighted the role or importance of microbes; compared to 936 (91%) of the 1020 articles in the UK (Supplementary Table S3). Across the 10 year period of the sample the emphasis in Australian newspaper articles has been on infections and the burden of disease caused by resistant microbes. Whereas since 2014, in the UK, much greater attention has been paid to the capacity of microbes to resist antibiotics and medical treatment, as the government introduced regulatory and research and development funding reforms (outlined in the 2013 UK Five Year AMR Strategy [23]) and began to drive global efforts to mitigate the risks and impacts of AMR—including championing the 2015 WHO Global Action Plan [56] (Figure 1). This dramatic change in public representations of the problem of AMR reframed public discourse in the UK to focus much more closely on its causes rather than its outcomes—even as UK health authorities were managing large outbreaks of antibiotic resistant infections. Arguably, the effect of framing problems around causes is to draw greater attention to the mechanisms and processes that promote different consequences, setting the stage for presentation and consideration of the potential for intervention and the opportunity for things to happen differently (Figure 2).

Against this background it was notable that antibiotics are mentioned in 327 (55%) of the 594 articles in the Australian sample and 430 (42%) of the 1020 in the UK sample (Supplementary Table S3). In Australian newspapers representations of antibiotics as being powerful therapeutic tools, misused, and prone to resistance are employed in relatively equal proportions, although in recent years the use of these frames is gradually declining. Instead antibiotics are increasingly being framed as lacking utility and being in short supply. Whereas in the UK descriptions in newspapers of antibiotics as being misused and prone to resistance are increasing (See Supplementary Figure S2).

### 3.2. Antibiotic Resistant Microbes That Are Most Prominent in Australian and UK Newspapers

**Figure 3:**
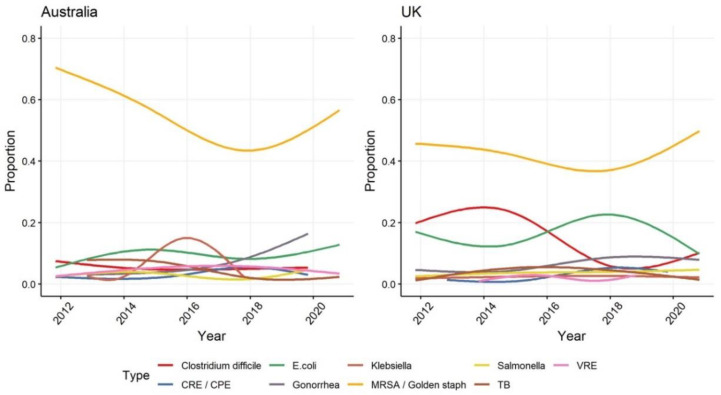
The nine most mentioned types of microbes in Australian and UK newspaper coverage about AMR.

Of the 594 articles in the Australian sample 410 (69%) mentioned at least one type of microbe compared to 820 (80%) of the 1020 articles in the UK. The list of the nine most mentioned microbes in Australia and the UK are identical. The microbes on these lists were all mentioned more than 10 times across the 10 years in both Australian and UK newspapers. Unsurprisingly, given the prominence of the superbug MRSA/Golden staph in the international recognition of AMR as a critical public health problem [5,58], coverage in both settings is dominated by accounts of the causes, impacts or solutions of these types of infections (46% of articles in Australia and 56% in the UK). The greater representation of other microbes such as *Clostridium difficile* (*C. diff*) and *Escherichia coli* (*E. coli*) in UK media sample was a consequence of the epidemiological scale of and political interest in specific impacts of outbreaks of these microbes during the sample period. As noted above, between 2000 and 2015, *C. diff* outbreaks in hospitals in the UK became a long-running national news story and political crises. Blame was ascribed to local failures of hospital trusts and staff to implement government legislated infection control codes of practice, and perceptions of falling levels of funding having impacts on standards of care in the National Health Service [53,59]. UK media attention changed to *E. coli* mid-decade as foodborne outbreaks and concerns about antibiotic use in agriculture became more prominent in both public discourse and scientific discussions [60,61]. Mentions of antibiotic resistant Gonorrhoea steadily increased in newspaper reports in both settings, providing a rare example where the community are being represented as having a key role in controlling outbreaks. In the UK, the government’s international leadership on combatting AMR and the real-world impacts of hospital and community AMR outbreaks has prompted greater media attention about the increasing resistance of microbes to treatment. In contrast, in Australia, where outbreaks of *C. diff* and *E. coli* were at a much smaller scale or had been rapidly controlled, the focus of media reporting has remained on the communicability of the disease and its consequences for individuals—with less focus on and need for causal explanations (Figure 3).

### 3.3. Newspaper Framings of the Reason Why Members of the Public Should Care about AMR

**Figure 4:**
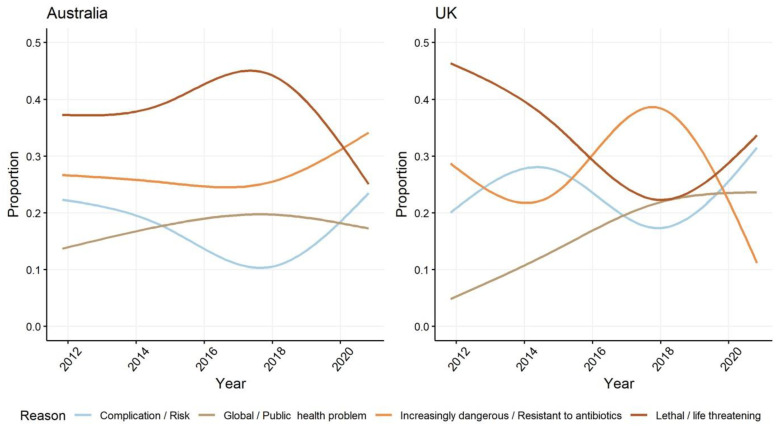
Proportion of different framings in Australian and UK newspapers of the reason why members of the public should care about AMR.

At least one reason why readers should care about the problem of AMR is provided in 89% of articles in the Australian newspaper sample and 86% in the UK (Supplementary Table S3). Early in the decade, media coverage of AMR in both settings emphasised the individual consequences of life-threatening disease. While the focus on the individual impacts increased mid-decade in Australia, at the same time, the emphasis in media coverage in the UK began to be placed on the causes and increasing level of risk posed by AMR. The initiation of an increased focus on the impacts of AMR on individual health in Australian media corresponds with the near simultaneous release and public dissemination of Australia’s First National Antimicrobial Resistance Strategy 2015–19 [26] and the WHO Global Action Plan [56] in 2015 (Figure 1). In recent years, as addressing AMR has become embedded in UK and Australian national policies, media representations of the threat to life posed by AMR have declined (compared to their starting point in 2010) in both settings studied. Instead, discussion of AMR escalation has become increasing prominent in Australia, as have framings in both settings of AMR as a complication, such as to be an unwanted but anticipated consequence of being infected with a treatment resistant microbe (Figure 4).

### 3.4. Newspaper Framings of Who and What Is Most at Risk from AMR

A group are identified as being at particular risk of AMR in 85% of the articles in the Australia sample, compared to 88% of articles in the UK (Supplementary Table S3). In both settings AMR is increasingly portrayed as being a general risk faced by the community, rather than something that is a risk to people who are subject to other healthcare needs. Notably, the emphasis placed on the risk to these two groups has changed in both media samples across the decade. While attention to healthcare associated infections and in hospitals has declined in Australia, in the UK AMR has been much more closely associated with healthcare settings. The impacts of AMR on Indigenous and Torres Strait Islander communities only began to be given media attention in 2017 with reporting of sporadic outbreaks of Gonorrhoea in the Northern Territory and the concerns of health authorities about the rates of rheumatic heart disease in children (Supplementary Figure S3).

**Figure 5:**
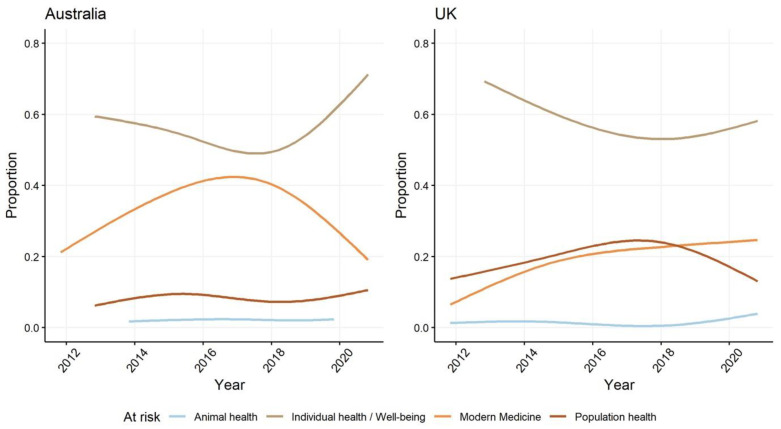
Proportion of different framings in Australian and UK newspapers of who and what is most at risk from AMR.

The most frequently described AMR-related risk within Australian and UK newspaper coverage is the loss of individual health and wellbeing, followed by the loss in effectiveness of the practice of modern medicine (Figure 5). Of these two representations of what is at risk, the non-lethal impacts on individuals are once again becoming more prominent in both Australian and UK newspaper coverage. Reporting on health issues in newspapers typically privileges the implications for individuals over description of societal impacts and their relationship to a broader social and political context [62,63]. After the burst of national and international policy activity in the first half of the decade drew greater public attention to the threat posed by AMR to health systems and populations (Figure 1), reporting in both setting appears to be returning to a style of journalism that individualises the problem or issue [63].

After a peak mid-decade, representations of AMR endangering the practice of high-technology modern medicine in Australian media are declining. In contrast the trend in the UK is a steady increase in the prominence of representations of the threat posed to the practice of modern medicine. Both media samples have an overwhelming focus on the risks to the health of individuals, but greater prominence is afforded in UK newspapers to representations of the impacts of AMR on the health of populations. The difference between the prominence of population health concerns in the two settings is likely to be a consequence of the frequent use of future mortality figures and projected death rates in newspaper reporting of the UK government’s international advocacy of AMR as a critical global health issue. Despite the widespread use of antimicrobials in agricultural industries, the impacts of AMR on animal health are rarely reported in the media in either study setting. Discussion of the risks to human health from antibiotic use in agriculture have been present throughout the period sampled in the UK. Despite the importance of One Health approaches in policy making in both jurisdictions [6], this has only recently been raised as a concern in reporting in Australia (Figure 5).

### 3.5. Newspaper Framings of Who or What Is Responsible for Causing the Problem of AMR

In Australian media coverage 520 of the 594 (87%) articles identify at least one key cause for the problem of AMR, which is a higher proportion than the 810 of 1020 (79%) of articles in the UK (Supplementary Table S3). Microbial evolution and antibiotic misuse in healthcare were identified as the key drivers or causes of AMR but the emphasis given to each is very different in newspapers in each study setting. For instance, in Australia attention is increasingly being drawn to the way in which microbes evolve to develop resistance on exposure to antibiotics, while the role of healthcare providers in promoting inappropriate antibiotic use is increasingly being downplayed. The opposite is happening in UK media coverage with greater attention being given to the importance of antibiotic misuse in healthcare, and less being afforded to the evolutionary consequences of these activities.

**Figure 6:**
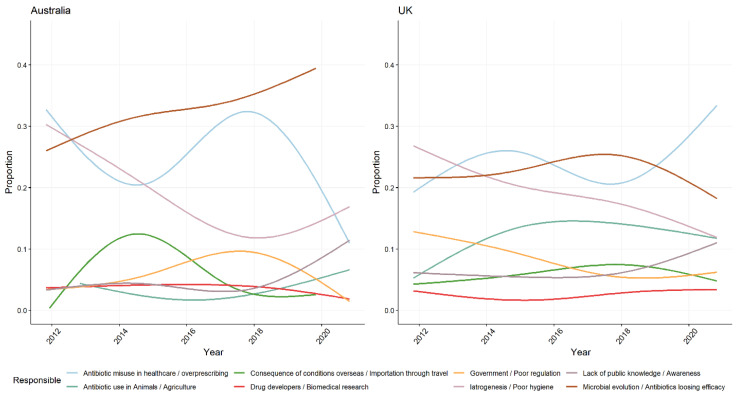
Proportion of different framings in Australian and UK newspapers of who and what is responsible for causing AMR.

In contrast to the UK, in newspaper coverage in Australia antibiotic overuse and microbial evolution are not consistently portrayed as being causally-connected. Discussion of social and economic drivers was much more prominent in the UK sample in the later part of the decade—including the influence of patient pressure on GP decision-making. Concomitantly, representations of the role of poor hygiene and iatrogenesis as being drivers of AMR have declined, after being previously identified foci for concerted action by the UK government due to the number and scale of community acquired MRSA cases and hospital based *C. diff* outbreaks at the time. Representations of the role of public in mitigating the drivers of AMR are infrequent in both settings despite being a stated priority in all Australian [26,30], UK [23,29] and WHO [56] AMR action plans [40,64] (Figure 6).

### 3.6. Newspaper Framings of Possible Solutions to the Problem of AMR

**Figure 7:**
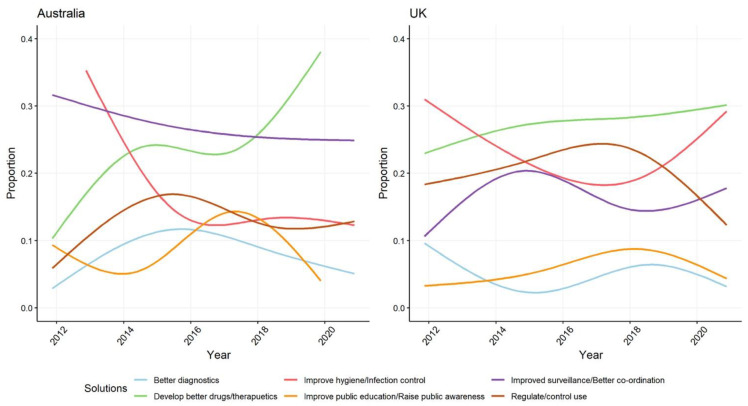
Proportion of different framings in Australian and UK newspapers of possible solutions to the problem of AMR.

Across the decade covered by our analysis, Australian media presents potential solutions to the problem of AMR less frequently than in the UK—discussion of solutions appears in 382 (64%) of Australian articles compared to 820 (80%) of those from the UK (Supplementary Table S3). In both settings, increasing emphasis is being given in media reports about the need to develop better drugs and therapeutics, but especially in Australia where the use of this frame has recently rapidly escalated. As the focus on therapeutic innovation in news coverage has increased in both settings, representations of measures to control or regulate antibiotic use to mitigate AMR are now declining. Suggestions involving greater regulation are in decline in both Australia and UK—even as regulatory action is largely absent from Australia’s 2013 and 2020 National Action Plans [6,26,30]. This change has been more rapid in the UK where antibiotic prescribing/use is now much more tightly controlled in healthcare and agriculture following a series of regulatory reforms earlier in the decade. In both settings, discussion of the idea that educating the public will lead to more appropriate antibiotic use has become less prominent. Perhaps because of the significant real-world impacts of *C. diff* and *E. coli* outbreaks, media coverage in the UK has included greater discussion of other potential measures such as improving surveillance, infection control, and hygiene (Figure 7).

### 3.7. Newspaper Representations of Who Is Responsible for Fixing the Problem of AMR

**Figure 8:**
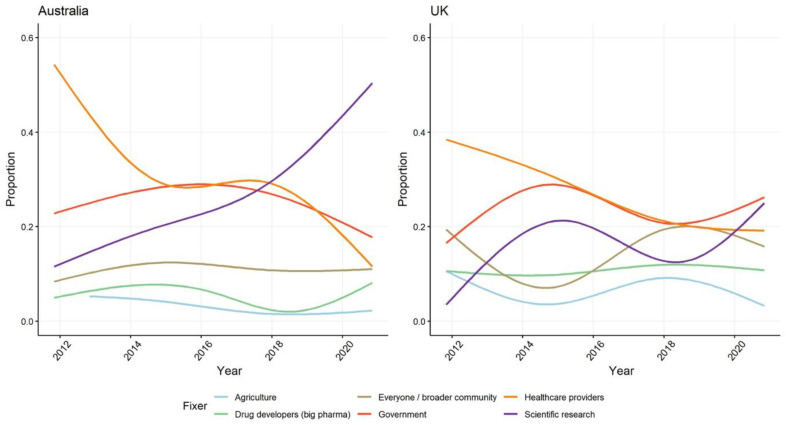
Proportion of different framings in Australian and UK newspapers of who is responsible for fixing the problem of AMR.

In the Australian newspaper sample, 437 of the articles (73%) point to a group as being responsible for fixing the problem of AMR. In the UK 802 of articles (80%) do the same (Supplementary Table S3). Across the span of the decade healthcare providers and scientific researchers are both emphasised as having an important role to play in attenuating the risks of AMR, but the trend in coverage, particularly in Australia, is to emphasise the potential for scientific discovery to solve the AMR problem. It is notable that in both media samples that by mid-decade, interviews with scientific researchers on AMR began to feature much more prominently; whereas, the proportion of articles with interviews with infectious disease experts or clinicians began to decline (Supplementary Figure S4). The greater prominence given to scientists and the potential of scientific discovery occurred slightly later in Australia but the trend is that the media presence of scientific researchers as experts on AMR is rapidly increasing. The current emphasis on scientists and technological solutions over prescribers and behaviour change is the opposite of how newspaper coverage in Australia ascribed primary responsibility for acting against AMR to healthcare providers a decade ago. Another key difference is that in the UK healthcare providers continue to be given a key role and responsibility for addressing AMR. Appeals for public action have not been as prominent in Australian newspaper coverage as in the UK (Figure 8). More broadly, media coverage in the UK ascribes a greater role to government action than in Australia—reflecting the extent to which politicians in the UK have taken public positions and been at the forefront of calls for global action against AMR.

## 4. Discussion

Our findings indicate there is much greater variability in the frames used in newspapers in the UK than Australia, with the former showing many more clear inflections in the trends of several of the framing categories—especially in the period between 2014 and 2016. Acknowledging it can be difficult to disentangle cause and effect regarding events, policy reforms and media interest, the increasing variability and volatility in the framing of AMR in UK newspapers corresponds to a period of rapid reform in how AMR policy in this jurisdiction was determined and implemented. From early in the decade the approach taken to AMR in the UK became much more regulatory, placing a greater emphasis on developing and using an evidence base to direct related policies and practices [6,27]. In contrast representation of the problem of AMR in Australian newspapers between 2010 and 2020 was less crisis driven and volatile—reflecting greater quiescence and a much more business as usual approach. The relative stability of framings of AMR in Australia compared to the volatility in the UK is likely to be a consequence of the extent to which direct exposure to large scale AMR epidemics have served as ‘focusing events’ which has raised the public and political interest in the issue requiring a much more radical response from health authorities and government [65]. Direct experience also can be a substantial factor in how members of the public apprehend and respond to media content about a shared problem [66], such that positive framing of potential solutions can resonate more among those who have already been impacted by the issue. This opened up a ‘policy window’ in the UK [67] where alternative courses of actions could be explored, and greater public and political interest and policy activity that has driven the development of a suite of actionable solutions, reforms and agendas. These solutions, reforms and agendas need to be communicated, explained and justified to publics and implicated stakeholders—therefore greater efforts at public communication in the UK and greater attention and variability in media representations of AMR-related issues.

The differences in newspaper framing of AMR in Australia and the UK points to the likely importance of events and material and local impacts to contextualise and provide impetus for practical measures to address otherwise abstract and existential risks [65]. There is experimental and empirical evidence that when news media messages are framed positively, they are seen as being less credible [68,69]. The theory is people learn to expect traditional news media to be credible and mainly negative such that an association between ‘bad news’ and credibility is built. In UK newspapers, the trend is that framings of AMR as life-threatening, as a risk or complication for individuals accessing healthcare, and as a product of over-prescribing are all increasing. This emphasis corresponds with the UK government’s most recent 5 year plan [29] which has an explicit focus on supporting clinicians to prescribe antibiotics appropriately. Compared to the reductions achieved within agricultural industries, the previous 5 year plan failed to substantially reduce antibiotic use in the community. The recent rapid increase in the focus in the UK on the potential for more catastrophic outcomes for individuals from AMR risks may also be a product of the sensationalism that characterises reporting in tabloid newspapers in the UK [70,71]. In comparison to Australia, AMR is consistently much more of a ‘bad news’ story than in the UK where recent targets for antibiotic use reductions have not been met, and health authorities and hospital trusts have struggled to contain significant outbreaks. So far Australia has not had a significant set of AMR ‘focusing events’ or ‘bad news’ stories—as resistance rates in key microbes remain comparatively low, even as community use of antibiotics is among the highest in the OEC.D. [72].

In both media depictions and policy discourses around health risks, how a problem is framed underpins public awareness and understanding, while also guiding and influencing the quality of the strategies and actions taken to address it [73,74,75]. Governments can employ a broad range of policy options to address AMR risks and impacts beyond public awareness campaigns and guidelines [20]. Policy responses in the UK have been oriented around centralised institutions with clear economic framework and accountability mechanisms. It is therefore notable that even though the use of different frames in coverage in UK newspapers varies considerably across the decade, causes, consequences and solutions to AMR are all represented in about 80% of the articles in the sample (Supplementary Table S3). In direct contrast AMR causes and consequences (87%) are much more frequently represented than AMR solutions (64%) in Australian newspapers content. Against this background our analysis confirms and is consistent with the findings of Davis and colleagues [40] that the Australian public have increasingly been given the message that they have a limited role to play in preventing future catastrophe. Australian media representations are normalising the threat of AMR such that is being seen as every-day part of the risks entailed by routine medical interventions, and therefore something that could happen to anyone in the community. Even though previous analyses of AMR content in UK newspapers highlight the prominence of discursive elements that work to remove agency from ordinary members of the public [42], there is greater focus on the systems and structures that drive antibiotic use in this coverage. Compared to the UK, solutions to AMR that entail lowering antibiotic consumption through system and structural reforms are not featured as prominently in Australian media reporting. Compounding this the emphasis placed in Australian newspapers on microbial evolution over factors related to human behaviours and practices foregrounds natural process and downplays need for people and institutions to act differently.

The increasing prominence of scientific frames and medical scientists in AMR related public discourses in both study settings may also be a product of the increasing institutional impetus for them to communicate the importance of their work to the general public [76], and, more broadly, the high levels of trust the public has for scientists compared to politicians and government agencies [77]. At the beginning of the decade, newspapers in UK and Australia sought the expertise of and interviewed hospital trust spokespersons and clinicians, respectively (See Supplementary Figure S4). The increasing prominence of scientific research and scientists in media coverage of AMR in the later part of the decade stands in contrast to the findings of analyses of more controversial issues such as climate change and the global financial crisis, where those who appeared in the mainstream media to discuss solutions tended to be those who are most supportive of the system which created the problems [66]. Consistent with other studies of public understandings and discourses, the political economic structural conditions contributing to antibiotic use are downplayed much more in Australian newspaper coverage, as are the significance of the actions and inactions of the pharmaceutical industry and industrial livestock producers [18,19]. Experimental studies on the impact of news media reporting on public opinion about controversial issues indicate that unless alternative solutions are presented, the message is much more likely to be ignored or rejected [66]. Arguably, AMR has been much more controversial in the UK than in Australia. Whereas the greater stability in the framing of messages in Australian newspapers may mean that members of the Australian public could be more resistant to adjusting their views or changing their opinions on the basis of new information. Even though AMR is not as politically charged and controversial as climate change, the net effect is to position publics as being spectators who might experience individual impacts from a collective action problem, rather than as agents who possess the potential to drive social action and transformation [66,78].

This study has several limitations including that our analysis only included materials from Australian and UK newspapers rather than broadcasts from national and local radio, television and social media. However evidence suggests that print news reporting continues to serve as the original source for much of what is reported later in other media, influencing a wider audience than just newspaper readers [36,79]. The inclusion of more local newspapers in the Australian sample compared to the UK may have increased the frequency of survivor stories and representations of the individual impacts of AMR in the Australian sample—noting that there is greater stability in the trends in the more geographically heterogeneous newspapers in Australia when compared to the trends in framing AMR in the more homogenous newspaper formats and markets covered in the UK sample. However, we also note the consistency of the outcomes of our study with other analyses of AMR in Australian and UK news media [40,41,42,43,53]. Policy activity and political culture have been shown to influence media coverage of emerging problems [25,67], and vice versa, but the direction of this influence can be difficult to determine [57]. With regard to this, not all issues can feasibly be at the top of the policy agenda [80,81]. Nevertheless, the variations in the trends of different frames around AMR in UK news reporting indicate that there has been a greater level of debate in both public and political discourses about the issue. In the UK this has drawn a broader range of stakeholders such as, for example, agricultural industries into making public statements in both mainstream and farming media defending how they are reforming antimicrobial use practices and policies in order to better protect human health [82].

## 5. Conclusions

AMR has not been a significant enough issue in Australia to drive the same levels of political, public and stakeholder engagement as seen in the UK. Widespread experience of the real world consequences of AMR have prompted government, health authority and stakeholder actions, which is reflected in and amplified by news media coverage. These media representations do have a possible role in changing how the public respond to specific issues such as AMR, especially where these are linked to structural support and changes in procedures [18,66]. Because of the higher levels of political commitment and appetite for structural and practice reforms, this has happened to a greater extent in the UK than in Australia. This suggests that using news media as an instrument to raise public awareness about AMR is neither a straightforward exercise in public education or a panacea—not the least because of the complex nature of the problem. As our results show, the way in which newspapers frame the issue of AMR can vary markedly and this can either limit or empower members of the public to act to address the issue. As the uptake of social and digital news media continues to fragment publics and audiences, public health policy actors need to refine their messaging and develop new approaches to begin to reshape public discourses and build public trust to garner public support for reforms to the systems and structures that surround antibiotic use and better promote the active role that citizens can play in reducing the drivers of AMR [39,47].

## Data Availability

Data is available on request.

## Supplementary Materials

The following are available online at https://www.mdpi.com/article/10.3390/antibiotics10121432/s1, Figure S1: Supplementary Figure S1—PRISMA diagrams for the Australian and UK media samples, Figure S2 Attributes of Antibiotics, Figure S3 Proportion of different framings in Australian and UK newspapers of who is most at risk from AMR, Figure S4 Key spokesperson interviewed who can speak to the importance of AMR, Table S1: Timeline of Key National Policies and Reports on AMR in Australia and UK 2011–2020, Table S2: Coding Tree for data extraction in media analysis of antimicrobial resistance (AMR) in Australian and UK newspapers Table S3: Proportion of articles in the Australian (*n* = 594) and UK (*n* = 1020) newspaper sample that represent each dimension.
