# Supplementary material for: Changes in the Framing of Antimicrobial Resistance in Print Media in Australia and the United Kingdom (2011–2020): A Comparative Qualitative Content and Trends Analysis
Source: Antibiotics (Basel). 2021 Nov 23;10(12):1432. doi: 10.3390/antibiotics10121432 (PMC8698020; doi:10.3390/antibiotics10121432)

Supplementary Table S1: Timeline of Key National Policies and Reports on AMR in Australia and UK 2011-2020

Year	Policy name	Country	Key areas of focus and recommendations
2011	Annual Report of the Chief Medical Officer Volume Two, 2011 *Infections and the rise of antimicrobial resistance*, London: Department of Health [8]	United Kingdom	• Optimise therapy for individual patients<br>• Prevent antibiotic overuse, misuse and abuse<br>• Minimise development of resistance at patient and community levels<br>• Incentivise research and development improve therapies and diagnostic technologies
2013	Department of Health and Department for Environment and Rural Affairs. (2013). *UK Five Year Anti-microbial Resistance (AMR) Strategy, 2013-2018.* United Kingdom [23]	United Kingdom	• Improve knowledge and understanding of AMR<br>• Conserve and steward the effectiveness of existing treatments<br>• Stimulate the development of new antibiotics, diagnostics and novel therapies
2015	World Health Organization. (2015). Global action plan on antimicrobial resistance. Geneva, WHO [53]	World Health Organization	• Develop a 'One Health' response to AMR which entails a collaboration across all sectors<br>• Improve awareness and understanding of antimicrobial resistance<br>• Strengthen the knowledge and evidence base through surveillance and research<br>• Reduce the incidence of infection through sanitation, hygiene and infection prevention<br>• To optimize the use of antimicrobial medicines in human and animal health<br>• To develop the economic case for investment in new medicines, diagnostic tools, vaccines and other interventions
2015	Department of Health, & Department of Agriculture. (2015). *Responding to the threat of antimicrobial resistance: Australia's first national antimicrobial resistance strategy 2015-2019*. Canberra, Australia [26]	Australia	• Increasing awareness and understanding of AMR<br>• Implementation of effective AMR stewardship<br>• Develop nationally coordinated One Health surveillance of AMR and antimicrobial use<br>• Improve infection prevention and control<br>• Strengthen international partnerships.<br>• Establish responsibilities for governance levels
2016	O'Neill, J. (2014). *Antimicrobial resistance: tackling a crisis for the health and wealth of nations*. London, UK: The Review on Antimicrobial Resistance [11]	United Kingdom	• Promote global collaboration<br>• Raise public and professional awareness of AMR<br>• Reduce antimicrobial use in agriculture<br>• Improve hygiene and infection control<br>• Improve global surveillance systems<br>• Establish greater incentives for new diagnostics and therapies
2019	HM Government. *Tackling antimicrobial resistance 2019-2024 – the UK's five-year national action plan*. 2019 [29]	United Kingdom	• Build on structures established in previous five year plan.<br>• Reduce the need for and unintentional exposure to antimicrobials<br>• Optimize the use of antimicrobials<br>• Invest in innovation, supply and access to tackle AMR
2019	Department of Health and Social Care 2019, *Contained and Controlled: The*	United Kingdom	• Lower the burden of infections<br>• Provide safe and effective care to patients

Year	Policy name	Country	Key areas of focus and recommendations
	UK's 20-year vision for antimicrobial resistance, Department of Health and Social Care, UK [81]		• Optimize antimicrobial use and ensure good stewardship across all sectors<br>• Develop new therapies, vaccines, and interventions<br>• Demonstrate appropriate use and support sustainable supply and access
2020	Department of Health & Department of Agriculture, Water and the Environment 2020, *Australia's National Antimicrobial Resistance Strategy - 2020 and beyond,* Department of Health & Department of Agriculture, Water and the Environment, Canberra. [30]	Australia	• Bring extended change and build on the previous five year plan<br>• Establish clear governance for AMR initiatives<br>• Prevent and control infections and AMR spread<br>• Establish greater engagement in the combat against AMR<br>• Promote appropriate usage and stewardship practices<br>• Promote integrated surveillance and response to AMR and usage<br>• Establish a strong collaborative research agenda across all sectors<br>• Strengthen global collaboration and partnerships

Supplementary Table S2: Coding Tree for data extraction in media analysis of antimicrobial resistance (AMR) in Australian and UK newspapers

	Coding category	Coding choices	Notes on code application
1	The key features of AMR highlighted in the article	Infection	AMR represented as a particular type of infection
		Microbial	AMR represented as a property of microbes
		Resistance	AMR represented as an impediment to effective treatment
2	Types of microbes	Acinetobacter baumannii	*Acinetobacter baumannii*
		Bacillus circulans	*Bacillus circulans*
		Borreliosis	*Borrelia burgdorferi* / Lyme disease
		Campylobacter	*C. jejuni; C. coli.; C. lari; C. upsaliensis*
		Candida	*C. auris; C. albicans*
		Chlamydia	*C. trachomatis*
		Clostridium difficile	*C. diff*
		CRE/CPE	Carbapenem-resistant/producing *Enterobacterales* (CRE / CPE)
		Escherichia coli	*E. coli*
		Gonorrhea	*Neisseria gonorrhea*
		Klebsiella	*K. pneumoniae; K. oxytoca*
		Legionella	*L. pneumophila*
		MRSA / Golden staph	Multi-resistant *staphylococcus aureus*
		Mycoplasma genitalium	Mgen
		NDM-1	New Delhi metallo-beta-lactamase-*1*
		Non-tuberculous mycobacterium	*Non-tuberculous mycobacterium*
		Pseudomonas	*P. aeruginosa*
		Salmonella	Range of Salmonella species
		Shigella bacterai	*Shigella bacterai*
		Strep A	*Streptococcus pyogenes*
		Syphilis	*Treponema pallidum*
		Tuberculosis	*Mycobacterium tuberculosis*
		VRE	Vancomycin-resistant *Enterococcus*
3	Reason why the reader should care about AMR	Complication / Risk	AMR is represented as a potential unwanted consequence of another activity or action
		Global / Public health problem	AMR is represented as a problem for Global health, public health, or both
		Increasingly dangerous/Resistant to antibiotics	The threat posed by AMR is represented as escalating / increasing
		Lethal / Life-threatening	AMR represented as a something that can kill people
4	Who is most at risk from AMR	Animals	AMR is represented as a risk for nonhuman animals - particularly those used in agriculture
		Community	AMR is represented as a risk for people / everyone in the community
		Indigenous Community	AMR is represented as a risk for Indigenous people
		Sick people / Hospital patients	AMR is represented as a risk for people who are unwell, accessing healthcare, or both
		Vulnerable groups	AMR is represented as a risk for vulnerable groups in the broader community
5	What is at risk from AMR	Animal health	AMR is represented as a risk to the health/productivity of nonhuman animals
		Individual health / Wellbeing	AMR is a risk to the health and wellbeing of individuals
		Modern medicine	AMR is a risk to the practice and availability of modern high-tech medicine
		Population health	AMR is a risk to the health of human populations

6	Who/what responsible for causing AMR	Antibiotic misuse in healthcare / overprescribing	AMR is caused by antibiotic misuse / overuse in human healthcare
		Antibiotic use in Animals / Agriculture	AMR is caused by antibiotic use in animals / agricultural production
		Consequence of conditions overseas / Importation through travel	AMR is caused by actions overseas / is imported into the country
		Drug developers / Biomedical research	AMR is caused by a failure to develop new therapies and medicines
		Government / Poor regulation	AMR is caused by a failure to regulate antibiotic use appropriately
		Iatrogenesis / Poor hygiene	AMR is caused by avoidable breakdowns in infection control / poor hygiene
		Lack of public knowledge / Awareness	AMR is caused by a lack of public knowledge/awareness about AMR
		Microbial evolution / Antibiotics losing efficacy	AMR is caused by microbes evolving in response to exposure to antibiotics
7	Who is responsible for addressing/fixing AMR	Agriculture	Farmers, veterinarians & agricultural industries are responsible for fixing AMR
		Drug developers (Big pharma)	Drug developers (Big pharma) are responsible for fixing AMR
		Everyone / broader community	Everyone is the community has a role in fixing AMR
		Government	Governments are responsible for fixing AMR
		Healthcare providers	Healthcare providers are responsible for fixing AMR
		Scientific research	Scientific research is responsible for fixing AMR
8	Antibiotics are represented as	Drug / Powerful therapeutic tool	Antibiotics are represented as a drug / powerful therapeutic tool
		Increasingly in short supply	Antibiotics are represented as increasingly in short supply
		Lacking utility	Antibiotics are represented as lacking utility
		Misused	Antibiotics are represented as misused
		Prone to resistance	Antibiotics are represented as being prone to promoting microbial resistance
9	Type of expertise of interviewees for the article	Affected member of the public	Perspectives/experiences of people impacted by AMR are prominent in reporting
		Chief Health Officer / Chief Scientist / WHO Spokesperson	Perspectives/experiences of Chief Health Officer / Chief Scientist / WHO Spokespersons are prominent in reporting
		Health agency spokesperson / Hospital CEO	Perspectives/experiences of health agency spokespersons / hospital CEOs are prominent in reporting
		Health minister / Politician	Perspectives/experiences of Health ministers / Politicians are prominent in reporting
		Infectious disease expert / Clinician	Perspectives/experiences of infectious disease experts / clinicians are prominent in reporting
		Scientific researcher	Perspectives/experiences of scientific researchers are prominent in reporting

Supplementary Table S3: Proportion of articles in the Australian (n=594) and UK (n=1020) newspaper sample that represent each dimension

	Australia	UK
Microbial aspects of AMR are represented	96%	91%
Attributes of antibiotics are represented	55%	42%
Reader given at least one clear reason for AMR being important	89%	86%
Identifies at least one risk group and set of consequences for AMR	85%	88%
Identifies at least one cause or driver of AMR	87%	79%
Identifies at least one potential solution to the problem of AMR	64%	80%
Identifies at least one group responsible for developing/implementing solutions to AMR	73%	80%

Supplementary Figure S1 – PRISMA diagrams for the Australian and UK media samples

Search terms

"antimicrobial resistan*" OR "super bug*" OR "superbug*" OR "flesheating bacteria" OR "flesh eating bacteria" OR "flesh-eating bacteria" OR "golden staph*" OR "MRSA"

01/01/2011 to 31/12/2020. English Language, Location/Region=Australia Only

Source: Australian News Media sources only (newspapers)

Subject: Political and General news only

Search Results:

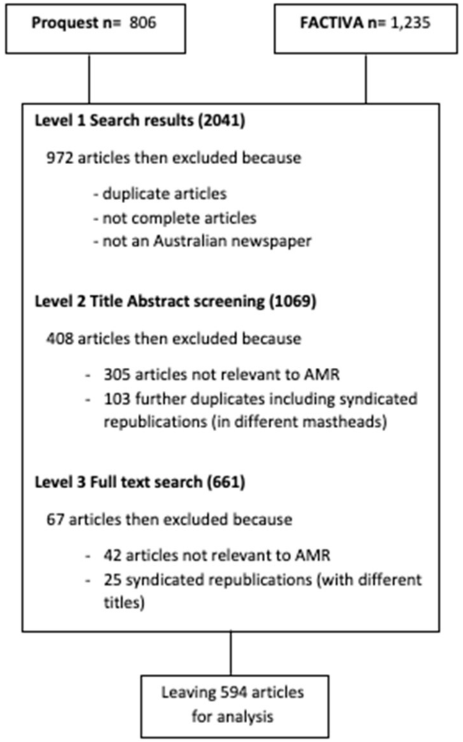

Search terms

"antimicrobial resistan*" OR "super bug*" OR "superbug*" OR "flesheating bacteria" OR "flesh eating bacteria" OR "flesh-eating bacteria" OR "golden staph*" OR "MRSA"

01/01/2011 to 31/12/2020. English Language, Location/Region=Australia Only

Source: UK News Print Media sources only (newspapers)

Subject: Political and General news only

Search Results:

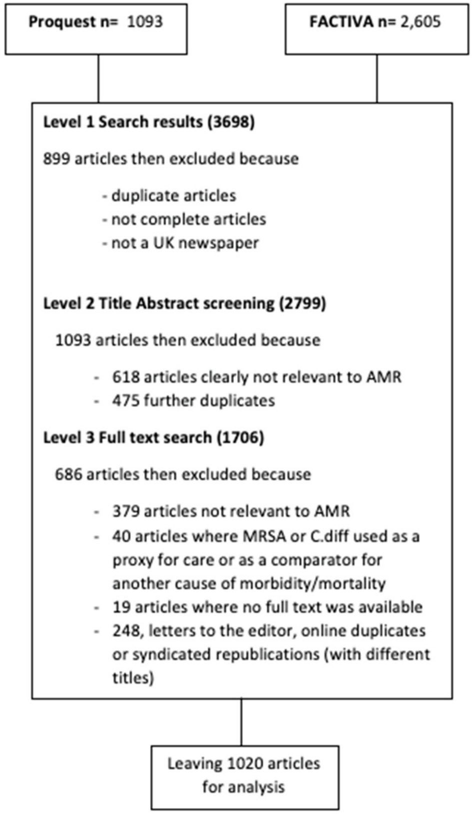

Supplementary Figure S2 *Attributes of*

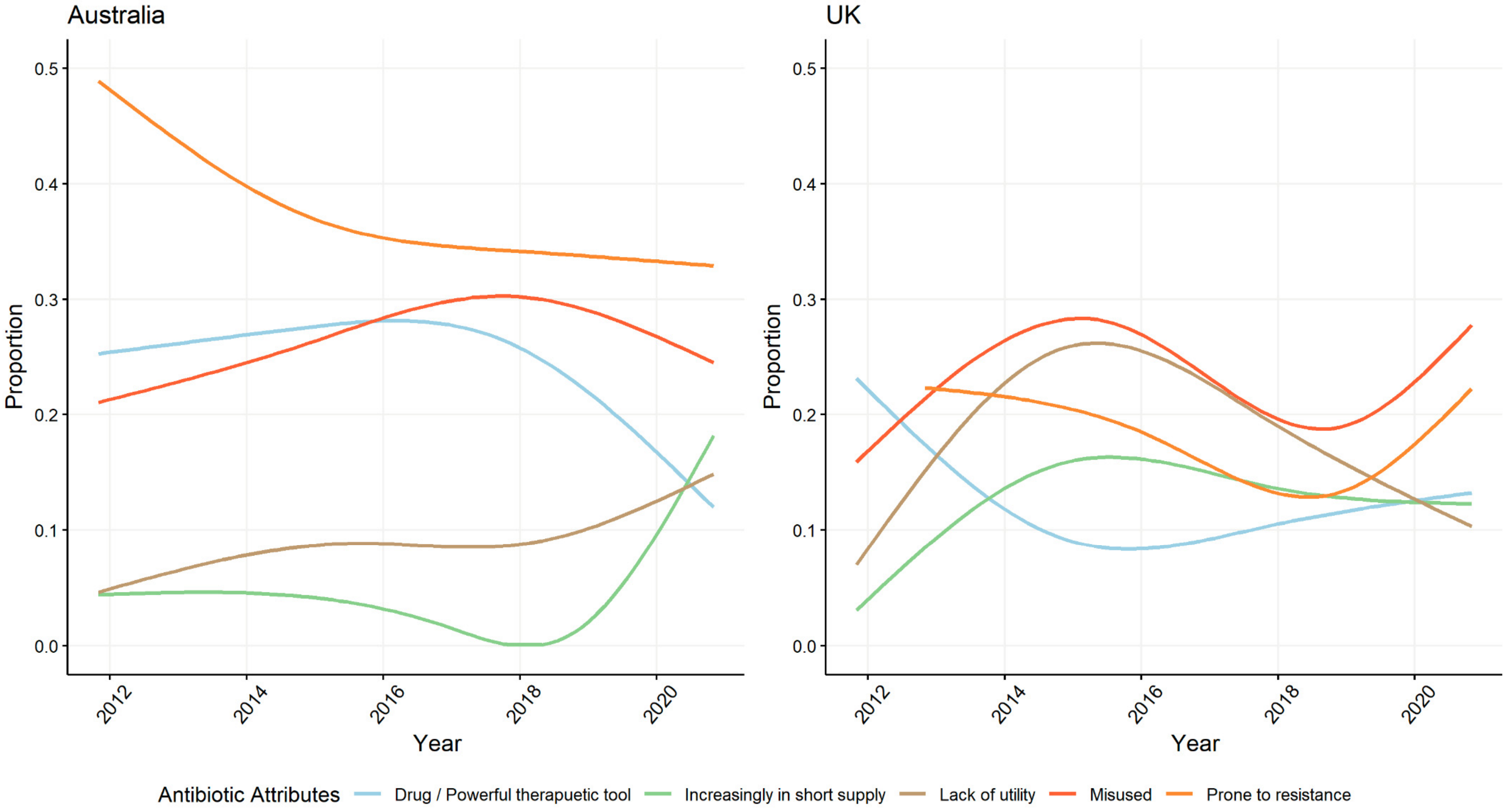

Supplementary Figure S3

Proportion of different framings in Australian and UK newspapers of who is most at risk from AMR

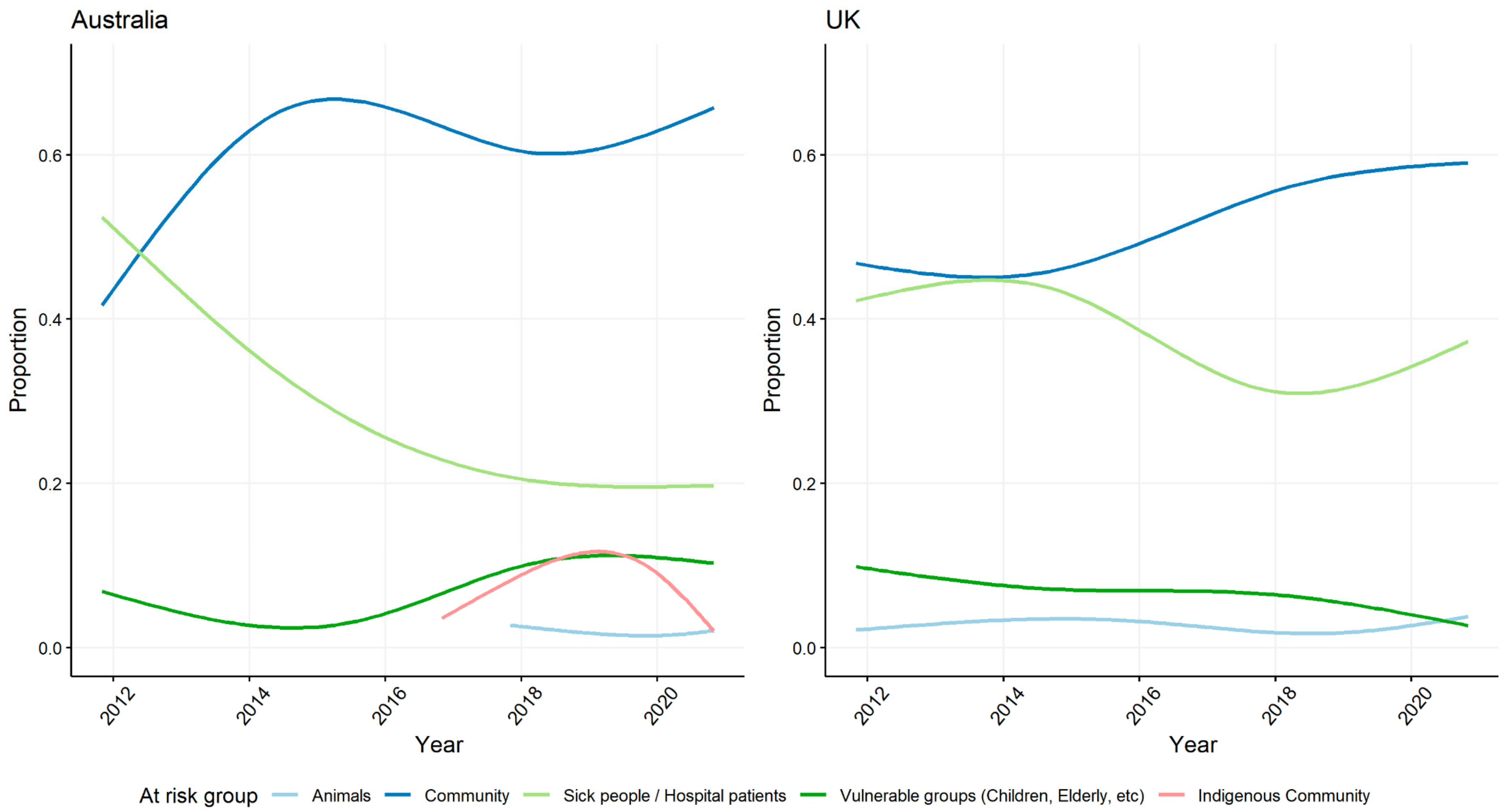

Supplementary Figure S4

Key spokesperson interviewed who can speak to the importance of AMR

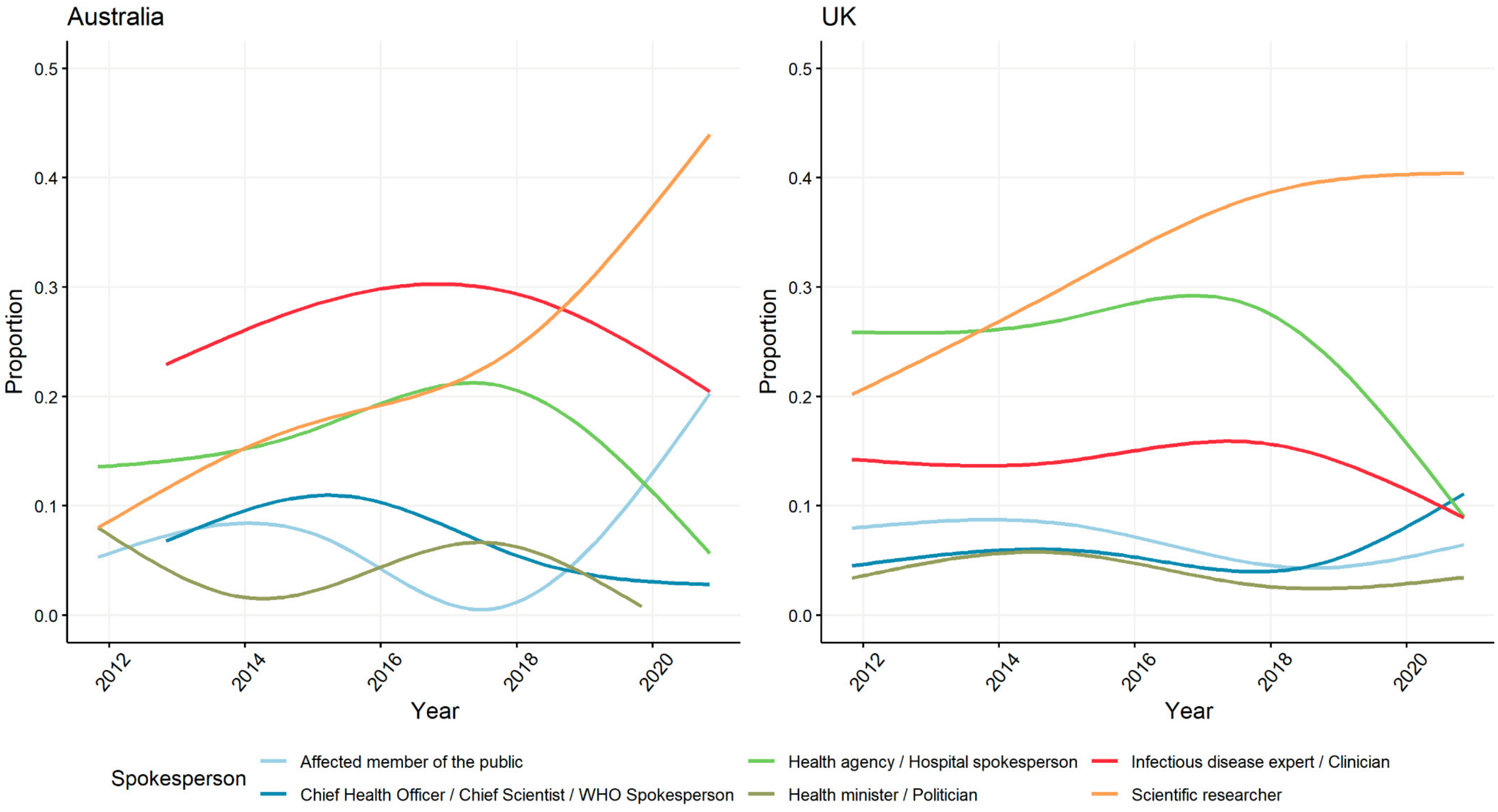

Supplement: Supplementary file 1 [file antibiotics-10-01432-s001.zip › antibiotics-1469955-supplementary.pdf]
